# Comparison of radiation dose and its correlates between coronary computed tomography angiography and invasive coronary angiography in Northeastern Thailand

**DOI:** 10.1186/s43044-022-00241-5

**Published:** 2022-01-25

**Authors:** Phatraporn Aupongkaroon, Pattarapong Makarawate, Narumol Chaosuwannakit

**Affiliations:** 1 Radiology Department, Faculty of Medicine, Khon Kaen University, Khon Kaen, 40000 Thailand; 2 Cardiology Unit, Internal Medicine Department, Faculty of Medicine, Khon Kaen University, Khon Kaen, Thailand

**Keywords:** Coronary CTA, CTA, Radiation dose, Coronary angiography

## Abstract

**Background:**

The number of coronary computed tomography angiography (CCTA) exams is steadily growing. A novel computed tomography (CT) system has been developed to increase image quality while lowering patient radiation. The radiation dose attributed to CCTA has received considerable attention, whereas the dose associated with invasive catheter angiography (ICA) has received less. This study aims to investigate the radiation exposure of CCTA in patients and compare it to ICA.

**Results:**

The mean effective dose of CCTA was 2.88 ± 0.85 mSv which was significantly lower than the mean effective dose of ICA (5.61 ± 0.55 mSv), *p* < 0.0001. The effective dose of CCTA correlated with the weight, height, and BMI, while the effective dose of ICA was associated with patient weight and BMI. The radiation exposure from CCTA has been considerably reduced over the last ten years by almost 2.5 folds. The mean radiation dose from the newer generation CT used in 2019 was significantly lower than that of the single-source CT in 2010 (2.88 ± 0.85 mSv vs. 7.15 ± 3.4 mSv, *p* < 0.001).

**Conclusions:**

CCTA allows evaluation of CAD with a significantly less effective radiation dose to patients than diagnostic ICA. There was a significant decrease in radiation dose from CCTA over time. Regular measurement of patient doses is an essential step to optimize exposure. It makes operators aware of their performance and allows comparisons with generally accepted practices.

## Background

**Fig. 1:**
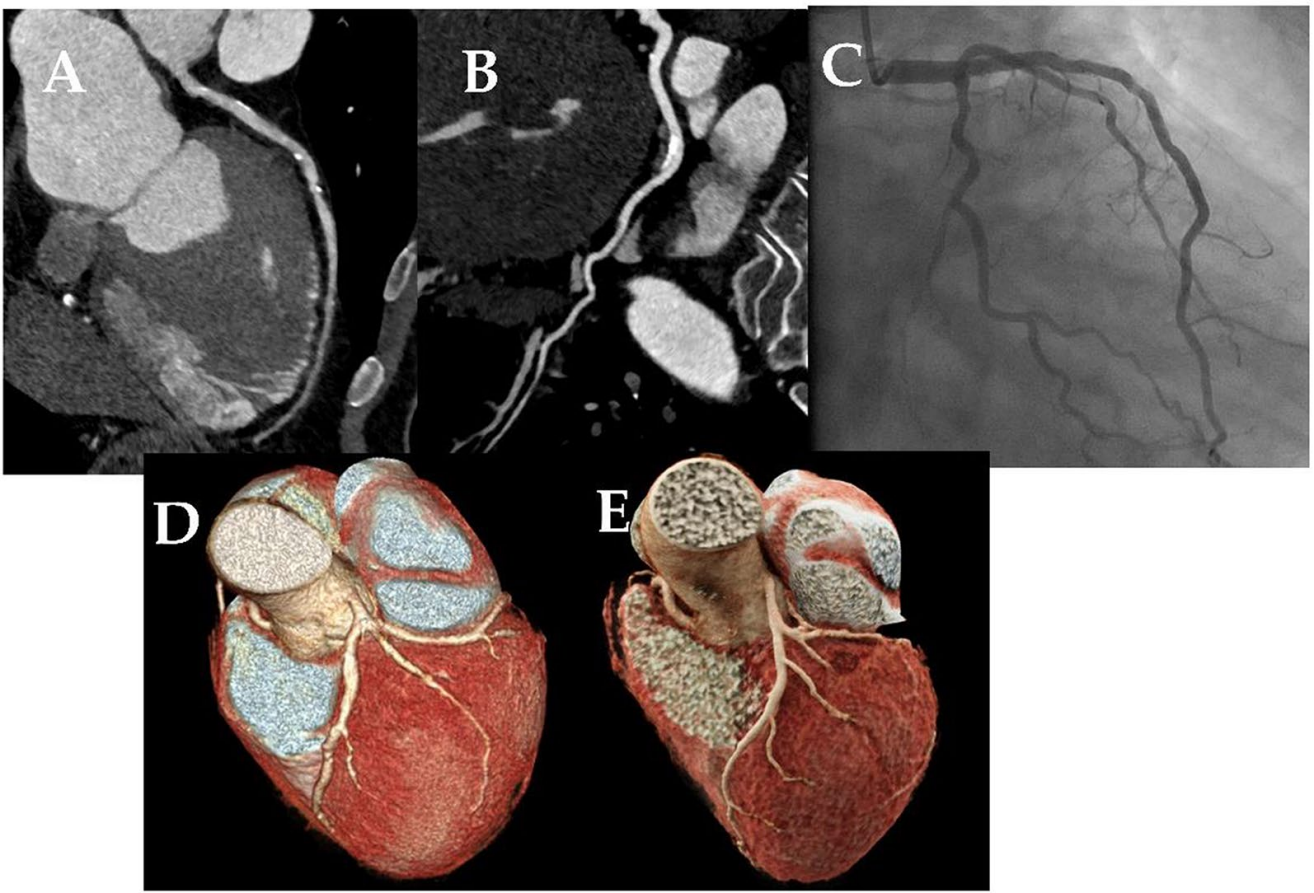
Coronary CTA images from single- source CT scanner (**A**, **D**), and dual-source CT scanner (**B**, **E**), and diagnostic invasive coronary angiography image (**C**)

The advancement of cardiac imaging modalities has resulted in significant improvements in the detection and treatment of cardiac disease in recent years. The coronary arteries have been visualized directly by coronary computed tomography angiography (CCTA) and invasive coronary angiography (ICA), which are the commonly used diagnostic modalities that involve ionizing radiation for assessing patients with possible coronary artery disease (CAD) (Fig. 1). The latter is considered the gold standard for the diagnosis of CAD [1, 2].

For imaging modalities that involve high doses or sensitive tissues in the primary radiation beam, the effective dose, absorbed dose, and organ doses are vital. Effective dose is a reasonable approximation of potential ionizing radiation damage and should be considered one parameter in determining the appropriateness of ionizing radiation examinations [2–4].

To determine the effective dose, we evaluated the computed tomography dose index (CTDI_vol_) and dose-length product (DLP) from CCTA [2–4].

To estimate the radiation dose for diagnostic ICA, we assessed the air kerma-area product (*P*_KA_) and expressed it in Gy.cm^2^, which is the recommended method to estimate the radiation dose for the patient in interventional cardiology [5].

This study aims to evaluate and compare the effective dose of CCTA and ICA and assess the correlation factors associated with high radiation dose and their advancement over time.

## Methods

### Patient population

We retrospectively reviewed patients’ radiation doses who underwent CCTA and diagnostic ICA at Khon Kaen University Hospital, the leading teaching hospital and advanced tertiary care institution in northeastern Thailand, between January 2019 to December 2019. Patient identification was made by reviewing our institution’s picture archiving and communication system (PACS) records data. The local Ethics Committee of Khon Kaen University, Thailand, reviewed and proved this study. The study was conducted according to the Declaration of Helsinki principles. All methods were performed following the relevant guidelines and regulations. The local Ethics Committee of Khon Kaen University also approved our investigation with a waiver of informed consent due to retrospective study design, and patient confidentiality was protected. Post-operative, post-revascularization, congenital heart disease patients, or the patients who underwent CCTA with increased field-of-view other than the standard scan range, and the missing radiation dose data in the picture archiving and communication system (PACS) were excluded from the present study.

### Coronary computed tomography angiography (CCTA)

Coronary CTA exams were done (Definition FLASH dual-source CT, Siemens Healthcare, Forchheim, Germany). The system consists of two X-ray tubes and two detectors positioned on a single gantry with a 90-degree angular offset. Two X-ray sources, double sampling by fast longitudinal modification of the focused point (Z-flying focal spot), rotation period 330 ms, automated tube voltage modulation were the CCTA scan standard protocol [6]. Patients were scanned while lying down. For the single-source CCTA exam, a 128-slice MDCT (Brilliance 128, Philips Healthcare, Netherland) used the following parameters: 128 × 0.6 collimation, 0.3 s rotation time, the pitch of 0.32, 120 kV tube voltage with ECG-triggering.

### Diagnostic invasive coronary angiography (ICA)

During the study period, 119 patients who underwent diagnostic ICA were recruited. The Philips Allura Xper biplane FD 10/10 (Eindhoven, Netherlands) was the two biplane angiocardiographic devices (double C-arc) in the catheterization lab at Khon Kaen University. The frame rate in normal fluoroscopy mode and digital cine acquisition was 15 frames per second. The system employs a sophisticated automatic dose control system for fluoroscopy automated spectrum beam filter selection.

### Definition and dosimetry

For each CCTA, the CTDI_vol_ and dose length product (DLP) were recorded. The patient dose data, CT dose index volume (CTDI_vol_), and dose length product (DLP) values, were extracted from the picture archiving and communication system (PACS). The Dose Length Product (DLP, units: mGy.cm) indicates the mean absorbed dose to the patient of each sequence in a CT exam and is calculated by multiplying CTDI_vol_ by the scan length. It measures the total CT examination’s mean effective dose to the patient [7, 8]. The CT monitor’s real-time CTDI_vol_ and DLP displays were collected in the PACS and retrospectively analyzed. For ICA, the air kerma-area product (*P*_KA_) meters quantify radiation dose in the unit and cumulative air kerma. The *P*_KA_ meters integrate exposure throughout the entire image field using an air ionization chamber installed in the X-ray assembly. The biplane’s total dose was determined in units of *P*_KA_ (Gy.cm^2^) [5]. The effective radiation dose was determined to mSv and compared imaging modalities based on current publications and manufacturer information. A factor of 0.014 mSv/mGy.cm was used to convert the CCTA dose-length product [9, 10], whereas 0.018 mSv/Gy.cm^2^ was used to convert the ICA air kerma-area product [5, 11]. The effective radiation dose was compared each year to assess the variation of radiation doses over time. In an attempt to discover predictors of dose change over time, all prospectively collected variables in the respective databases were analyzed.

### Statistical analysis

SPSS Statistics 17.0 for Windows was used to accomplish the statistical analysis. Unless otherwise stated, continuous variables are reported as mean and standard deviation, and categorical variables are presented as number (*n*) or frequency (percent). The nonparametric Mann–Whitney or Kruskal–Wallis tests were used to evaluate continuous variables. Spearman correlation coefficient was used to assess the correlation between the effective dose and patient characteristic factors. The chi-square test was used to compare frequency distributions. Two-sided *p *values of less than 0.05 were considered statistically significant.

## Results

**Fig. 2:**
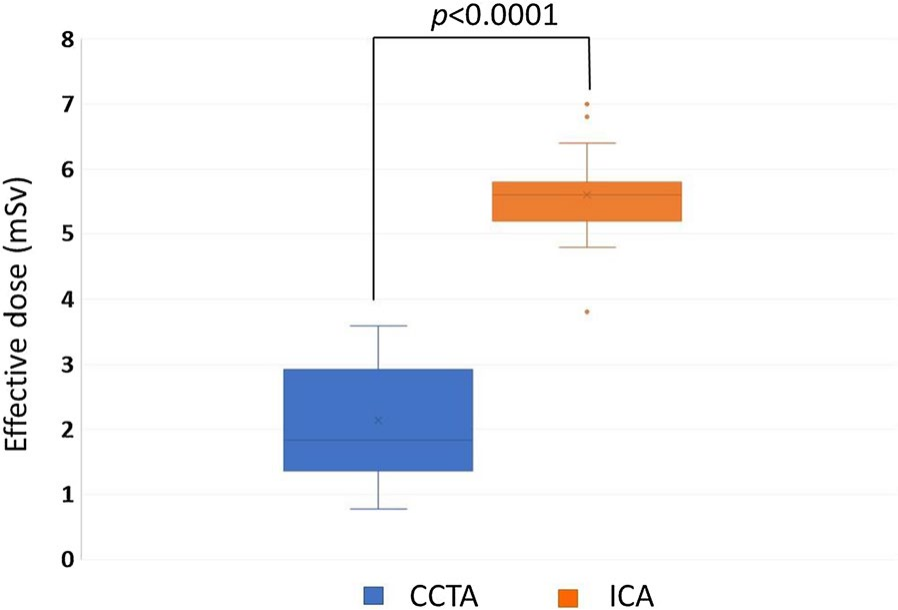
Box plots show that the mean effective doses for the coronary CTA (CCTA) were significantly lower than the mean effective dose for invasive coronary angiography (ICA)

During the retrospective study period, 406 CCTA studies and 119 diagnostic ICA were recruited. The mean effective dose of CCTA performed between January to December 2019 was 2.88 ± 0.85 mSv, significantly lower than the mean effective dose of diagnostic ICA *(*5.61 ± 0.55 mSv*), p* < 0.0001 (Table 1). For ICA, the mean *P*_KA_ was 55.2 ± 37.8 Gy.cm^2^, cumulative air Kerma was 782 ± 394.8 mGy, and the mean fluoroscopic time was 8.8 ± 10.9 min. The effective dose comparing between procedures by box plots is shown in Fig. 2.

**Table 1:** Radiation dose from coronary CTA (CCTA) and invasive diagnostic coronary angiography (ICA)

Parameters	CTA (*n* = 406)	ICA (*n* = 119)	*P* value (95% CI)
Effective dose (mSv), mean ± SD	2.88 ± 0.85	5.61 ± 0.55	< 0.0001* (2.43–3.02)
Gender, (male, %)	24 (30.7)	14 (35.9)	0.70 (− 17.09 to 32.28)
Age (years), mean ± SD	65.5 ± 9.6	65.8 ± 9.4	0.87 (− 3.40 to 4.0)
BMI (kg/m^2^), mean ± SD	23.4 ± 3.6	23.1 ± 3.4	0.66 (− 1.67 to 1.07)

*Statistically significant at the *p* value < 0.05

When we investigated the correlation between the effective dose and patient characteristics for CCTA, the results showed a strong linear relationship between the effective dose and body weight (*r* = 0.42, *p* < 0.001), BMI (*r* = 0.387, *p* < 0.001), and a good linear relationship between the effective dose and height (*r* = 0.27, *p* = 0.017), respectively (Table 2).

**Table 2:** Correlation between patient characteristics and effective dose of coronary CTA

	Correlation coefficient (*r*)	*P* value
Age	− 0.25	0.273
Weight	0.42	0.000129*
Height	0.27	0.017*
BMI	0.387	0.000465*

^*^Pearson correlation is significant at the *p* value < 0.05 [2-tailed]

For ICA, we found a poor linear relationship with no significant between the effective dose and patient age and height. The results also demonstrated an excellent linear relationship between the effective dose and BMI (*r* = 0.24, *p* = 0.0116) and a fair relationship between the effective dose and weight (*r* = 0.29, *p* = 0.04) as demonstrated in Table 3 for the Spearman correlation coefficient.

**Table 3:** Correlation between patient characteristics and effective dose of ICA

	Correlation coefficient (*r*)	*P* value
Age	− 0.04	0.808
Weight	0.29	0.04*
Height	0.18	0.27
BMI	0.24	0.0116*

*Pearson correlation is significant at the *p* value < 0.05 [2-tailed]

**Fig. 3:**
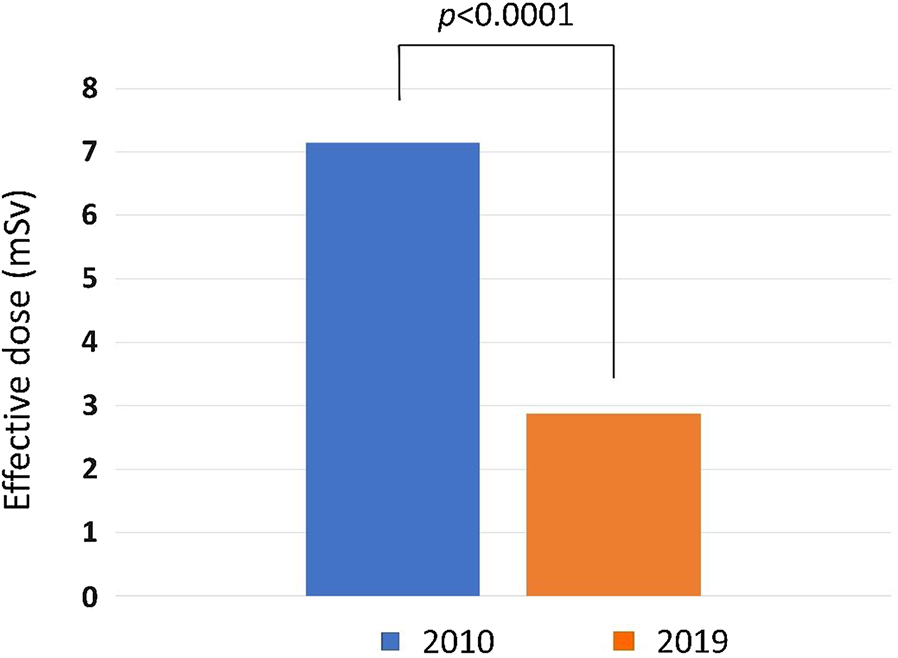
Box plots show that the mean effective doses for the coronary CTA (CCTA) performed in 2019 were significantly lower than the mean effective dose for CCTA in 2010

We have previously reported on the effective radiation dose associated with CCTA performed in 2010 in a single-center experience demonstrated the effective radiation dose of coronary CTA ranged from 2.8 to 11.5 mSv depended on different dose-saving techniques and heart rates, and the mean effective dose is 7.15 ± 3.4 mSv [12]. Compared to the present study, we documented a significant decrease in radiation dose by CCTA over time and identified the correlation of a higher radiation dose. The radiation exposure from CCTA has been considerably reduced over the last ten years by almost 2.5 folds. The mean CTDI_vol_ and DLP from the newer generation CT used in 2019–2020 was significantly lower than that of the single-source CT in 2010 (9.8 ± 2.7 mGy vs. 36.7 ± 7.8 mGy, *p* < 0.0001 and 188 ± 46 mGy.cm vs. 584 ± 98 mGy.cm, *p* < 0.0001) (Table 4; Fig. 3).

**Table 4:** Comparison of radiation dose and patient characteristics between dual-source CTA (DSCTA) in 2019 and single-source CTA (SSCTA) in 2010

Parameters	DSCTA (*n* = 406)	SSCTA (*n* = 233)	*P* value (95% CI)
Effective dose (mSv), mean ± SD	2.88 ± 0.85	7.15 ± 3.4	< 0.0001* (3.64–4.89)
Gender, (male,%)	24 (30.7)	9 (47.4)	0.14 (− 4.57 to 38.55)
Age (years), mean ± SD	65.5 ± 9.6	68.3 ± 10.2	0.06 (− 9.79 to − 0.60)
BMI (kg/m^2^), mean ± SD	23.4 ± 3.6	22.9 ± 5.5	0.58 (− 2.3 to 1.32)

*Statistically significant at the *p* value < 0.05

## Discussion

Our study discovered a wide range of effective radiation doses associated with routine cardiovascular diagnostic procedures. The radiation dose for diagnostic ICA was significantly higher, whereas the dose for CCTA was considerably lower. We also uncovered CCTA-related trends in the mean effective radiation dose and a relationship between radiation dose and certain clinical factors. There is a gross lack of information on radiation doses to patients in most Asian countries and the absence of reports from Thailand on this issue.

To our knowledge, the present study is the first investigation of comparing the patient radiation dose during various diagnostic cardiac procedures in Thailand. We hope it may stimulate interest in the area to benefit both patients and staff.

In Northeastern Thailand, this is the first report of radiation dose from diagnosis ICA utilizing the two biplane angiocardiographic devices (double C-arc). The results of this study will promote radiation dose optimization and benefits in both patients and staff.

We have previously reported on the effective radiation dose associated with CCTA performed in 2010 in a single-center experience demonstrated the effective whole-body dose of CCTA ranged from 2.8 to 11.5 mSv depended on different dose-saving techniques and heart rates. The mean effective dose is 7.15 ± 3.4 mSv. For patients in the prospective ECG-triggering *(*PT*)* group, the mean DLP was 184 ± 66 mGy.cm, resulting in an effective radiation dose per examination of 3.1 ± 1.1 mSv. In the retrospective (RG) group, the mean DLP was 501 ± 198 mGy cm, resulting in an effective radiation dose per examination of 8.5 ± 3.4 mSv. Compared to the present study, we documented a significant decrease in radiation dose by CCTA over time and identified the correlation of a higher radiation dose. The radiation exposure from CCTA has been considerably reduced over the last ten years by almost 2.5 folds. The mean radiation dose from the newer generation CT used in 2019 was significantly lower than that of the single-source CT in 2010, *p* < 0.0001. In 2010, the median effective dose for coronary CTA was 7.15 ± 3.4 mSv. Still, by 2019, it has dropped to 2.88 ± 0.85 mSv, resulting in a 59.7% reduction in radiation exposure or over 2.5 times reduction in the effective dose (*p* < 0.0001).

Notably, the number of non-diagnostic coronary CTAs did not rise over the study period, remaining below 3% in 2010 and 2019. As the number of detector slices increases and with faster gantry rotation speeds, the temporal and spatial resolutions improve cardiac imaging, consistent with the prior study by Liang et al. [13] that coronary CTA performed on dual-source CT results in better image quality lower radiation dose than single-slice CT.

On this concept, ionizing radiation procedures should be conducted with the “as low as reasonably achievable” philosophy in consideration, and clinicians ordering and conducting cardiac imaging diagnostic tests should be knowledgeable with the associated radiation doses and strategies for reducing them. The mean effective radiation dose we discovered for each exam corresponds with previous studies with the same dual-source CT scanner [14, 15]. Kosmala et al. [14] studied the radiation dose of CCTA with a third-generation dual-source scanner in a real-world patient population and demonstrated the median effective dose was 1.32 mSv for prospective sequence and 4.77 mSv for retrospective sequence.

Furthermore, we evaluated certain variables that influence the effective radiation dose delivered by these exams. Obese individuals received considerably greater mean effective radiation doses in all the exams investigated. This was especially true of CCTA and ICA. For CCTA, the effective radiation dose was correlated with higher weight, BMI, and height, demonstrated by another study [16]. For ICA, the effective radiation dose correlated with higher BMI and weight, in line with published data [17].

There are some limitations in the present study. Firstly, the current study is retrospective, single-center experience; hence, further prospective study with larger recruited patients should be considered.

Secondly, because of several technical advancements, the CCTA has significantly reduced radiation dose, which can currently be reached at the submillisievert level.

It’s important to remember that ICA has been following these developments, which were not included in this study. Because of decreased frame rates and magnification, appropriate collimation, and software solutions that interpolate virtual images between frames, ICA radiation exposure has been significantly reduced in the contemporary catheterization laboratory during the last decade.

Finally, the ICA registry’s higher radiation dose was also correlated to a higher number of positive and complexity of coronary artery disease. However, we did not analyze this. We can assume that the complexity of conditions needed more cine angiograms of the coronary arteries, with a consequent increase in the radiation dose used [18, 19].

## Conclusions

CCTA provides a comprehensive CAD assessment, yet it exposed patients to a substantially lower effective radiation dose than diagnostic ICA.

The radiation exposure from CCTA decreased significantly over ten years period.

Measuring patient doses on a regular schedule is critical for optimizing exposure.

It promotes operator awareness of their performance and allows for comparisons with generally accepted practices.

## Data Availability

None.

## Abbreviations

CCTA: Coronary computed tomography angiography
ICA: Invasive coronary angiography
CAD: Coronary artery disease
CTDI_vol_: Computed tomography dose index
DLP: Dose-length product
P_KA_: Air kerma-area product
PACS: Picture archiving and communication system
